# Efficacy of salmeterol/fluticasone propionate by GOLD stage of chronic obstructive pulmonary disease: analysis from the randomised, placebo-controlled TORCH study

**DOI:** 10.1186/1465-9921-10-59

**Published:** 2009-06-30

**Authors:** Christine R Jenkins, Paul W Jones, Peter MA Calverley, Bartolome Celli, Julie A Anderson, Gary T Ferguson, Julie C Yates, Lisa R Willits, Jörgen Vestbo

**Affiliations:** 1 Woolcock Institute of Medical Research, Sydney, Australia; 2 Division of Cardiac and Vascular Science, St George's, University of London, London, UK; 3 University Hospital, Liverpool, UK; 4 St Elizabeth's Medical Centre, Boston, USA; 5 GlaxoSmithKline (GSK), Stockley Park, UK; 6 Pulmonary Research Institute of Southeast Michigan, Livonia, USA; 7 GSK, Research Triangle Park, USA; 8 Wythenshawe Hospital, Manchester, UK; 9 Hvidovre Hospital, Hvidovre, Denmark

## Abstract

**Background:**

The efficacy of inhaled salmeterol plus fluticasone propionate (SFC) in patients with severe or very severe COPD is well documented. However, there are only limited data about the influence of GOLD severity staging on the effectiveness of SFC, particularly in patients with milder disease.

**Methods:**

TORCH was a 3-year, double-blind, placebo-controlled trial of 6112 patients with moderate/severe COPD with pre-bronchodilator FEV_1 _< 60% predicted (mean age 65 years, 76% male, mean 44% predicted FEV_1_, 43% current smokers). To understand the relative efficacy of SFC and its components by GOLD stages, we conducted a post-hoc analysis of the TORCH dataset using baseline post-bronchodilator FEV_1 _to segment patients into three groups: moderate COPD (GOLD stage II and above: ≥ 50%; n = 2156), severe COPD (GOLD stage III: 30% to < 50%; n = 3019) and very severe COPD (GOLD stage IV: < 30%; n = 937).

**Results:**

Compared with placebo, SFC improved post-bronchodilator FEV_1_: 101 ml (95% confidence interval [CI]: 71, 132) in GOLD stage II, 82 ml (95% CI: 60, 104) in GOLD stage III and 96 ml (95% CI: 54, 138) in GOLD stage IV patients, and reduced the rate of exacerbations: 31% (95% CI: 19, 40) in GOLD stage II, 26% (95% CI: 17, 34) in GOLD stage III and 14% (95% CI: -4, 29) in GOLD stage IV. SFC improved health status to a greater extent than other treatments regardless of baseline GOLD stage. Similarly, SFC reduced the risk of death by 33% (hazard ratio [HR] 0.67; 95% CI: 0.45, 0.98) for GOLD stage II, 5% (HR 0.95; 95% CI: 0.73, 1.24) for GOLD stage III, and 30% (HR 0.70; 95% CI: 0.47, 1.05) for GOLD stage IV. The rates of adverse events were similar across treatment arms and increased with disease severity. Overall, there was a higher incidence of pneumonia in the fluticasone propionate and SFC arms, compared with other treatments in all GOLD stages.

**Conclusion:**

In the TORCH study, SFC reduced moderate-to-severe exacerbations and improved health status and FEV_1 _across GOLD stages. Treatment with SFC may be associated with reduced mortality compared with placebo in patients with GOLD stage II disease. The effects were similar to those reported for the study as a whole. Thus, SFC is an effective treatment option for patients with GOLD stage II COPD.

**Trial registration:**

Clinicaltrial.gov registration NCT00268216; Study number: SCO30003

## Background

The last decade has seen a series of randomized controlled trials (RCTs) of pharmacological treatment which have provided a strong evidence base for the role of drug treatment in the management of chronic obstructive pulmonary disease (COPD) [1]. The efficacy of inhaled corticosteroid/long-acting β-agonist (ICS/LABA) combinations, including the salmeterol/fluticasone propionate combination (SFC), in COPD has been clearly shown for many clinically relevant outcomes including exacerbation frequency, rate of lung function decline and health status in patients with severe and very severe COPD (GOLD stages III and IV) [2-4]. To date there has been a paucity of information about the effectiveness of these agents in patients with GOLD stage II COPD, with the robustness of any clinical conclusions drawn being limited by the relatively small size of the subgroups reported [5].

Previous RCTs examining treatment effects with these drugs recruited patients entirely or predominantly from GOLD stages III and IV [6-9]. These data contributed to COPD treatment guidelines recommending the use of ICS/LABA combinations to reduce the frequency and severity of exacerbations and improving lung function and health status in patients with more severe COPD (forced expiratory volume in one second [FEV_1_] < 50% predicted) and a history of exacerbations. The absence of RCT data applicable to patients with GOLD stage II COPD can now be redressed through analysis of the results in patients with milder disease in recent large trials [2,10].

The TORCH study is the largest trial of pharmacotherapy ever undertaken in COPD. It randomized over 6000 patients, and investigated the effects of SFC, salmeterol (SAL), fluticasone propionate (FP) and placebo on mortality, lung function, exacerbations and quality of life in patients with COPD. The study included patients with a pre-bronchodilator FEV_1 _of less than 60% predicted irrespective of their prior exacerbation history [11]. As GOLD stages of severity are defined by the post-bronchodilator FEV_1_, a substantial proportion of TORCH participants had GOLD stage II disease. The TORCH data therefore provide a unique opportunity to analyse the clinical efficacy and adverse events (AEs) profile of SFC and its components (SAL and FP) in patients at different stages of COPD. In this post-hoc analysis we have focussed on the effects of SFC on mortality, exacerbations, lung function and quality of life by GOLD stage, with particular emphasis on patients with diagnosed GOLD stage II disease.

## Methods

Full details of the TORCH methodology have been published previously [2,11].

### Patients

Current or former smokers with at least a 10-pack-year history, aged between 40 and 80 years, with a confirmed diagnosis of COPD and pre-bronchodilator FEV_1 _less than 60% of the predicted value were enrolled in the TORCH study. In addition, patients were required to show less than 10% reversibility (as a percentage of predicted FEV_1_) to 400 μg salbutamol and a FEV_1_/forced vital capacity (FVC) ratio of 0.70 or less. Patients were excluded if they had a diagnosis of asthma or other non-COPD respiratory disorder, any other condition likely to cause death within 3 years, previous lung volume reduction surgery and/or lung transplantation, a requirement for oxygen therapy for at least 12 hours per day, current use of oral corticosteroid therapy, or experienced an exacerbation requiring systemic oral corticosteroid therapy and/or hospitalization during the run-in period. All patients gave written informed consent. The study was approved by local ethics review committees and was conducted in accordance with the Declaration of Helsinki and Good Clinical Practice guidelines.

### Study design

The TORCH study design has been described in detail in previous publications [2,11]. Briefly, 6112 patients at 439 centres in 42 countries were included in the efficacy analyses and 6184 patients at 444 centres were included in the safety analyses. Patients were randomized using a double-blind parallel group design to receive twice-daily administration of SAL 50 μg, FP 500 μg, SFC 50 μg/500 μg, or placebo for 3 years. The primary efficacy outcome was all-cause mortality over 3 years, regardless of whether patients withdrew from the study early. Secondary outcomes were exacerbation rate, health status, lung function and AEs while on treatment.

### GOLD stage analysis

For this post-hoc analysis, baseline post-bronchodilator FEV_1 _was used to group patients into GOLD-stage categories. At baseline, the highest of three acceptable measurements of FEV_1 _was recorded 30 minutes after inhalation of 400 μg salbutamol via metered-dose inhaler and Volumatic spacer (ELLIPSE at US centres). The GOLD stages are categorized as follows: stage II corresponds to post-bronchodilator FEV_1 _50% to < 80% predicted, stage III to 30% to < 50% predicted, and stage IV to < 30% predicted [1]. In this post-hoc analysis of TORCH, the GOLD stage II category included 28 patients (five, two, nine and 12 in the placebo, SAL, FP and SFC treatment arms, respectively) with a post-bronchodilator FEV_1 _≥ 80% (GOLD stage I).

### Statistical analysis

Time to all-cause mortality was analyzed using the log-rank test, stratified by smoking status. Exacerbation rates were analyzed using a generalized linear model (assuming the Negative Binomial distribution, to account for patient variability), adjusted for age, gender, body mass index (BMI), baseline FEV_1_, previous exacerbations, region and smoking status. Quality of life was determined using the St George's Respiratory Questionnaire (SGRQ). Total SGRQ scores were analyzed as changes from baseline values using repeated measures analysis of covariance (ANCOVA), using the covariates age, gender, BMI, baseline FEV_1_, baseline SGRQ, region and smoking status. Post-bronchodilator FEV_1 _was analyzed using repeated measures ANCOVA with covariates of age, gender, BMI, baseline FEV_1_, region and smoking status. The rate of decline in FEV_1 _was analyzed using a random coefficients model adjusted for the same covariates, with random patient effects. AEs and serious AEs (SAEs) were coded using the Medical Dictionary for Regulatory Affairs (MedDRA, version 8.1) and summarized by treatment arm. Time to first pneumonia analyzed using the log-rank test, stratified by smoking status and was compared across treatment arms using Kaplan-Meier estimates. Pneumonia rates were expressed as per 1000 treatment years, by dividing the total number of events by the total time on treatment in years, then multiplying by 1000.

To determine whether the treatment effects were consistent across severity groups, the interaction of treatment by severity group was tested for each endpoint.

## Results

### Demographics

Baseline characteristics were similar across groups when stratified by GOLD stage (Table 1). The main differences between the groups were that the GOLD stage IV group had a higher proportion of males (83% versus 76% and 72% in the GOLD stage III and II groups), contained more former smokers (66% versus 57% and 53% in the GOLD stage III and II groups) and experienced more exacerbations requiring oral corticosteroids or antibiotics in the year prior to the study (mean of 1.3 versus 1.0 and 0.9 in the GOLD stage III and II groups). Baseline SGRQ total scores were higher with increasing disease severity, defined spirometrically. Mean reversibility within each GOLD stage was less than 5% of the predicted FEV_1 _(Table 1). A total of 16 patients violated the entry criteria and had reversibility 10–15% (six on placebo, three on SAL, six on FP and one on SFC). Of patients categorized as GOLD stage II, most had post-bronchodilator FEV_1 _at baseline of 50% to < 60%, however there were 796 patients (37%) with FEV_1 _≥ 60% (Table 2).

**Table 1:** Demographic and baseline characteristics by GOLD stage*

**Variable**	**Stage IV** **(< 30% predicted)** **(n = 937)**	**Stage III** **(30% to < 50% predicted)** **(n = 3019)**	**Stage II** **(≥ 50% predicted)** **(n = 2156)**	**Total population** **(n = 6112)**
age, mean (years)	64.2 ± 7.8	65.4 ± 8.1	64.9 ± 8.7	65.0 ± 8.3
male (%)	83	76	72	76
BMI, mean (kg/m^2^)	23.5 ± 4.9	25.1 ± 5.0	26.6 ± 5.2	25.4 ± 5.2
smoking status: current (%)	34	43	47	43
exacerbations				
number requiring antibiotics and/or oral corticosteroids, mean	1.3 ± 1.5	1.0 ± 1.4	0.9 ± 1.2	1.0 ± 1.3
number requiring hospitalization, mean	0.4 ± 0.7	0.3 ± 0.7	0.2 ± 0.5	0.2 ± 0.6
post-bronchodilator FEV_1_, mean (ml)	704 ± 160	1108 ± 263	1616 ± 399	1226 ± 443
% predicted post-bronchodilator FEV_1_, mean	24.6 ± 4.0	40.1 ± 5.7	58.8 ± 7.4	44.3 ± 13.4
SGRQ score, mean	(n = 730)	(n = 2460)	(n = 1761)	(n = 4951)
total score	56.5 ± 15.0	50.0 ± 16.5	45.4 ± 17.7	49.3 ± 17.1
symptoms score	67.0 ± 18.0	63.5 ± 19.3	60.3 ± 21.0	62.9 ± 19.9
activity score	73.4 ± 16.7	64.1 ± 19.0	57.1 ± 20.6	63.0 ± 20.0
impact score	43.6 ± 18.6	37.7 ± 18.8	33.6 ± 19.6	37.1 ± 19.3
reversibility as % of predicted FEV_1_, mean	2.5 ± 3.2	3.6 ± 3.6	4.3 ± 4.0	3.7 ± 3.7

*Plus-minus values are mean ± standard deviation

**Table 2:** Post-bronchodilator FEV_1 _% predicted at baseline

**FEV_1_, % predicted, n (%)**	**placebo** (n = 1524)	**SAL** (n = 1521)	**FP** (n = 1534)	**SFC** (n = 1533)	**total** (n = 6112)
< 30%	214 (14)	260 (17)	220 (14)	243 (16)	937 (15)
30% to < 50%	775 (51)	739 (49)	777 (51)	728 (47)	3019 (49)
50% to < 60%	347 (23)	335 (22)	329 (21)	349 (23)	1360 (22)
60% to < 70%	148 (10)	160 (11)	165 (11)	173 (11)	646 (11)
70% to < 80%	35 (2)	25 (2)	34 (2)	28 (2)	122 (2)
≥ 80%	5 (< 1)	2 (< 1)	9 (< 1)	12 (< 1)	28 (< 1)

### Withdrawal

**Figure 1:**
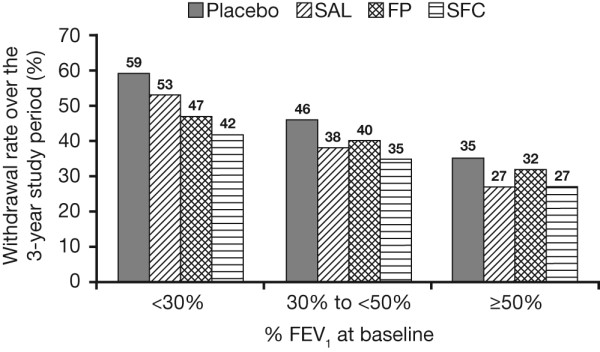
**Rate of withdrawal of patients over the 3-years duration of the study by baseline post-bronchodilator FEV_1 _% predicted**.

Increasing severity by GOLD staging was associated with a higher probability of withdrawal over the 3-year study. More patients withdrew in the placebo arm compared with the SFC arm in all severity stages, and the lowest rate of withdrawals across all treatments was in patients in GOLD stage II (Figure 1).

### Mortality

**Figure 2:**
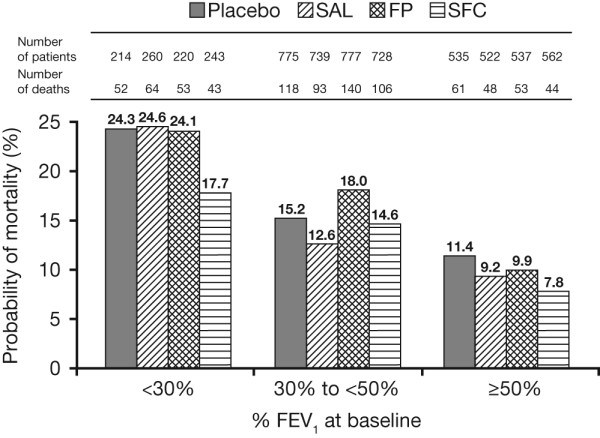
**All-cause mortality by baseline post-bronchodilator FEV_1 _% predicted**.

In GOLD stage II, the risk of death was reduced by 33% (HR 0.67; 95% CI: 0.45, 0.98; 11.4% of the patients died on placebo compared with 7.8% on SFC). The absolute risk reduction was 3.6%. The risk of death was reduced by 5% (HR 0.95; 95% CI: 0.73, 1.24) in GOLD stage III patients and by 30% (HR 0.70; 95% CI: 0.47, 1.05) in GOLD stage IV patients (Figure 2).

The effects of SAL and FP on the probability of death versus placebo or SFC were generally similar across GOLD stages (Figure 2).

### Moderate/severe exacerbation rates

**Figure 3:**
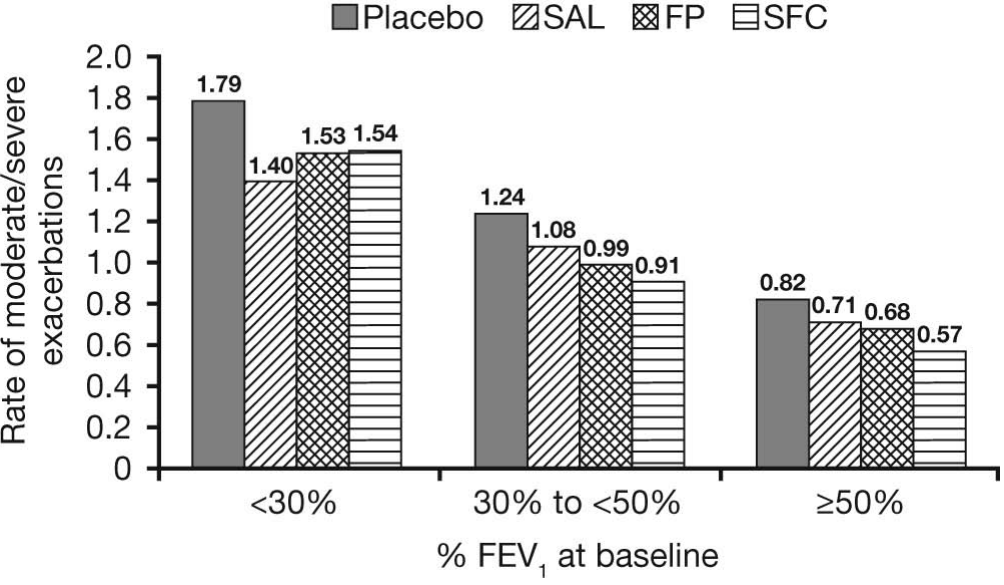
**Exacerbation rate by baseline post-bronchodilator FEV_1 _% predicted**.

SFC reduced the annual rate of exacerbations by 31% (CI: 19, 40) compared with placebo (mean of 0.57/year in SFC versus 0.82/year in placebo) in patients with GOLD stage II COPD. Patients with GOLD stage III and IV COPD also experienced a reduction in exacerbations. Compared with placebo, SFC reduced the number of exacerbations in patients with GOLD stage III COPD by 26% (CI: 17, 34) per year (mean of 0.91/year for SFC versus 1.24/year for placebo). In patients with GOLD stage IV COPD, SFC reduced exacerbations by 14% (CI: -4, 29) per year versus placebo (mean of 1.54/year for SFC versus 1.79/year for placebo) (Figure 3).

### FEV_1_

**Figure 4:**
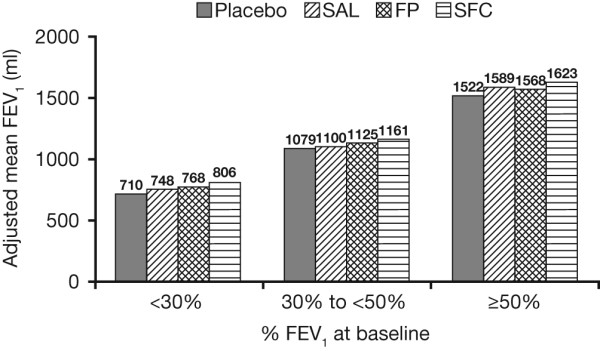
**Adjusted mean FEV_1 _over 3 years by baseline post-bronchodilator FEV_1 _% predicted**.

Improvements in FEV_1 _with SFC versus placebo were 101 ml (95% CI: 71, 132) in GOLD stage II patients, 82 ml (95% CI: 60, 104) in GOLD stage III patients and 96 ml (95% CI: 54, 138) in GOLD stage IV patients (Figure 4).

**Figure 5:**
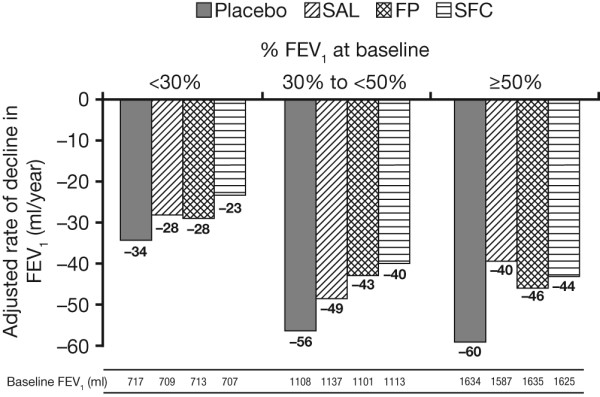
**Rate of decline in FEV_1 _by baseline post-bronchodilator FEV_1 _% predicted**.

The reduction in the rate of decline in FEV_1 _with SFC versus placebo was 16 ml/year (95% CI: 0, 32) in GOLD stage II patients, 16 ml/year (95% CI: 5, 28) in GOLD stage III patients and 11 ml/year (95% CI: -8, 30) in GOLD stage IV patients (Figure 5).

### Health status

**Figure 6:**
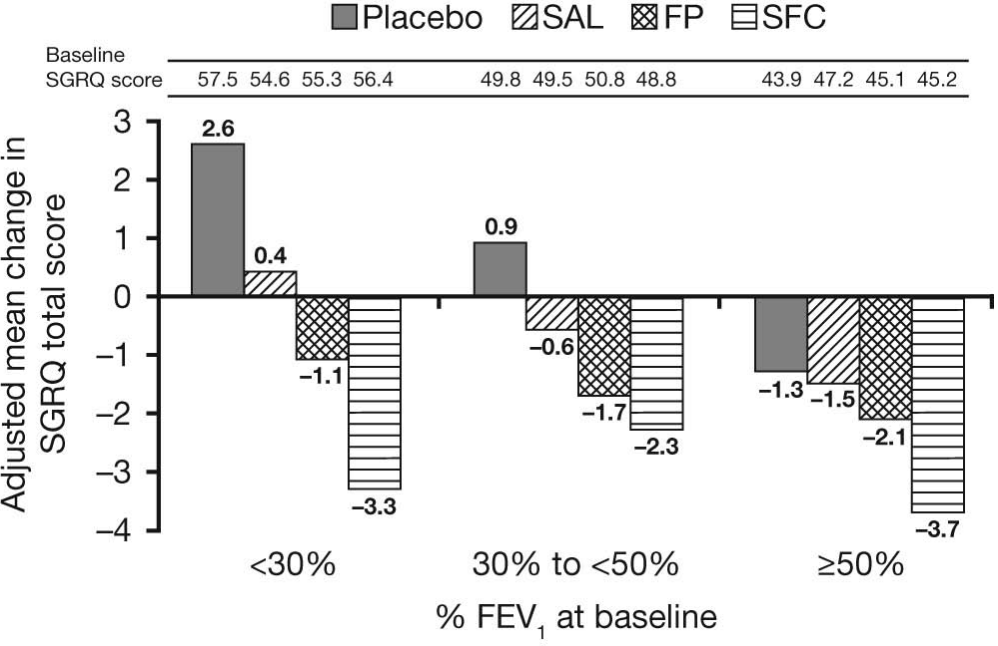
**Improvement (reduction) in SGRQ vs placebo by baseline post-bronchodilator FEV_1 _% predicted**.

The greatest improvement relative to placebo was observed in those patients with more severe disease treated with SFC (Figure 6). The difference in adjusted mean change in SGRQ for SFC versus placebo was -2.3 (95% CI: -4.0, -0.7) in GOLD stage II, -3.3 (95% CI: -4.7, -1.9) in GOLD stage III and -5.9 (95% CI: -8.7, -3.0) in GOLD stage IV. A numerical trend to greater improvement in SGRQ with worsening GOLD stage was noted with all active treatments, however this was not statistically significant.

### Treatment interaction

There was no evidence of a difference in treatment effect across the GOLD stages on all-cause mortality (p = 0.402 for the interaction test), exacerbations (p = 0.254), post-bronchodilator FEV_1 _(p = 0.298), rate of decline in FEV_1 _(p = 0.830) or SGRQ (p = 0.321).

### Safety

Consistent with the results from the original analysis, the incidence of any AE was similar across the treatment arms, irrespective of GOLD stage. The incidence of SAEs and fatal AEs was also similar across treatment arms, and increased with increasing disease severity (Table 3). The most frequently reported AE, irrespective of GOLD stage, was an exacerbation of COPD.

**Table 3:** Incidence of adverse events by post bronchodilator % predicted FEV_1_*

**Variable**	**Placebo** (n = 1544)	**SAL** (n = 1542)	**FP** (n = 1552)	**SFC** (n = 1546)
**FEV_1 _< 30% predicted**				
n	215	261	223	246
any AE, n (%)	198 (92)	241 (92)	212 (95)	230 (93)
serious AEs, n (%)	108 (50)	142 (54)	129 (58)	134 (54)
fatal AEs, n (%)	26 (12)	35 (13)	35 (16)	25 (10)
**FEV_1 _30% to < 50% predicted**				
n	786	750	785	735
any AE, n (%)	717 (91)	669 (89)	702 (89)	664 (90)
serious AEs, n (%)	322 (41)	306 (41)	357 (45)	327 (44)
fatal AEs, n (%)	70 (9)	62 (8)	87 (11)	62 (8)
**FEV_1 _≥ 50% predicted**				
n	543	531	544	565
any AE, n (%)	470 (87)	471 (89)	481 (88)	487 (86)
serious AEs, n (%)	197 (36)	174 (33)	169 (31)	198 (35)
fatal AEs, n (%)	37 (7)	29 (5)	38 (7)	27 (5)

*Safety population (n = 6184)

**Figure 7:**
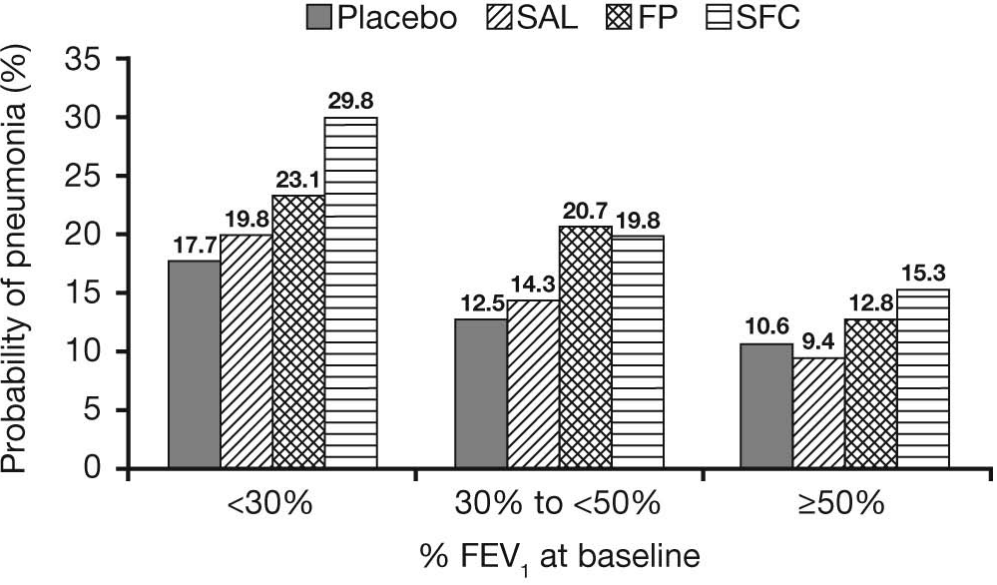
Probability* of pneumonia by 156 weeks by baseline post-bronchodilator FEV_1 _% predicted. *Kaplan-Meier probability.

The incidence of pneumonia increased with disease severity, irrespective of treatment. The probability of pneumonia as an AE was increased in patients receiving ICS-containing therapy (SFC, FP) compared with those patients not receiving ICS (SAL, placebo) in all GOLD stages (Table 4; Figure 7). When investigating treatment interactions for time to first pneumonia, there was no evidence of treatment differences across GOLD stages (p = 0.402).

**Table 4:** Pneumonia adverse events by post-bronchodilator FEV_1 _% predicted at baseline*

	**Placebo** (n = 1544)	**SAL** (n = 1542)	**FP** (n = 1552)	**SFC** (n = 1546)
**FEV_1_< 30% predicted**				
number of patients	215	261	223	246
total treatment exposure (yrs)	378	511	487	546
number of events	28	44	55	89
rate‡	74	86	113	163
**FEV_1 _30% to < 50% predicted**				
number of patients	786	750	785	735
total treatment exposure (yrs)	1626	1686	1787	1752
number of events	87	90	171	156
rate‡	54	53	96	89
**FEV_1 _≥ 50% predicted**				
number of patients	543	531	544	565
total treatment exposure (yrs)	1275	1334	1281	1402
number of events	55	48	74	79
rate‡	43	36	58	56

*Safety population (n = 6184)

‡Rate per thousand treatment years, calculated as (events × 1000/total treatment exposure)

## Discussion

Large prospective randomized clinical trials are designed to report their pre-specified outcomes in the recruited population. However, clinicians are also interested in whether treatment responses vary within specific subsets of patients which, in the case of COPD, have been defined in terms of the post-bronchodilator FEV_1 _thresholds used in the GOLD guidelines. Indeed guidelines require this type of post-hoc analysis since they encourage more targeted therapy to specific patient groups. Based on the size of studies like TORCH and UPLIFT [10], there is reasonable power to conduct exploratory post-hoc analysis of secondary outcomes. In the case of TORCH, where approximately one-third of the TORCH study population fell into the GOLD stage II category, the present post-hoc analysis demonstrates that SFC improved SGRQ, reduced exacerbations and improved lung function when compared with placebo. SFC was also associated with reduced mortality in GOLD stage II patients, compared with placebo.

TORCH recruited patients with a history of COPD, reversibility to salbutamol of < 10% of predicted FEV_1 _and excluded patients with a diagnosis of asthma. Reversibility to bronchodilation has been shown to be variable within COPD patients and the presence or absence of reversibility on a single test is not an important criterion to predict response to ICS [12]. A very small number of patients in TORCH (16 patients) were protocol violators on the reversibility entry criterion.

The primary purpose of the TORCH trial was to determine whether SFC reduced all-cause mortality compared with placebo treatment (effectively regular short-acting bronchodilator therapy). As discussed elsewhere [2], TORCH was probably underpowered to show this difference in a 4-arm study design and so particular caution is needed when interpreting mortality data between GOLD stage subgroups in this post-hoc analysis. However, while patients showed an increased risk of dying as their baseline spirometry worsened, there was a lower mortality with SFC treatment even in those with GOLD stage II disease. Clearly this finding should be confirmed by further prospective studies.

As expected, the burden of disease increased with severity regardless of the endpoint measured (mortality, exacerbations, FEV_1_, and AEs). All subgroup analyses should be treated with caution. However, treatment to prevent exacerbations seemed just as effective whatever the GOLD stage. The exacerbation frequency on placebo was lowest in GOLD stage II, but was not negligible with a rate of 0.82 events per year. We observed the same proportionate reduction in events with SFC therapy in GOLD stage II patients suggesting that treatment would be worthwhile for these patients. Deterioration in health status was seen over 3 years in the placebo arm in patients with GOLD stages III and IV, but those in GOLD II showed a small improvement. SFC improved health status by approximately the same amount from baseline in all three GOLD stages. In patients with more severe disease, the main effect of SFC appears to be to slow the rate of progression relative to placebo. However, it is clear that patients in GOLD stage II do have better health status when treated with SFC compared with placebo, with a change of over two units in total score maintained over the 3 years of study.

The treatment effects on post-bronchodilator FEV_1 _followed a similar pattern across all GOLD stages. The rate of decline in FEV_1 _tended to be slightly lower in GOLD stage IV than in stage II. SFC reduced the rate of FEV_1 _decline by 16 ml/year versus placebo in GOLD stage II patients versus 11 ml/year in stage IV patients which is comparable to the result reported in the overall population [13].

In our study, the incidence and severity of AEs was generally comparable across treatment arms, regardless of GOLD stage. Increased incidence of pneumonia has previously been reported with the use of ICS-containing therapy [2] and whilst this was observed, the probability of pneumonia with SFC in GOLD stage II patients was lower than that observed in the overall population. Further study is required however, to determine the exact mechanisms involved. There were too few on-treatment deaths from pneumonia to analyze these by GOLD stage.

It is important to note that, although GOLD stage II is defined by a post-bronchodilator FEV_1 _> 50% but < 80% predicted [1], the upper limit for eligibility to the TORCH study was pre-bronchodilator FEV_1 _< 60% predicted; therefore most patients would be expected to fall into the more severe end of stage II COPD, which was indeed found to be the case. The majority of patients who were diagnosed with GOLD stage II COPD had a baseline post-bronchodilator FEV_1 _of 50% to < 60% (1360 patients [64%], while 646 patients [30%] had a FEV_1 _60% to < 70% and only 122 [6%] had a FEV_1 _70% to < 80%). This distribution appears to be similar to the FEV_1 _% predicted distribution of stage II COPD patients from a 2005–2007 UK General Practice Research Database population-based cohort study [GSK Worldwide Epidemiology (WEUSKOP2207) Final study report, May 2009. Data on file] and the demographics of the TORCH GOLD stage II population is also similar to that of the UPLIFT clinical trial [10]. The majority of patients were also sufficiently symptomatic, as indicated by high baseline SGRQ total score, to have presented and been diagnosed with COPD, most likely as a result of exacerbations.

This analysis provides key information on pharmacotherapy in patients presenting with milder COPD and such data are lacking in the current literature and guidelines. Calverley and colleagues [5] performed a similar post-hoc analysis of the TRISTAN trial, but divided patients into FEV_1 _< 50% predicted and ≥ 50% predicted and explored treatment differences by severity as a continuous variable. Lung function was found to improve with active treatment, irrespective of FEV_1_, with greatest improvements reported for patients treated with SFC. In contrast to the findings reported here, only patients with more severe disease reported significantly reduced exacerbations; however health status and breathlessness both improved with active treatment irrespective of FEV_1_. The present findings provide further evidence that SFC can be used in patients with milder COPD.

Inevitably a post-hoc analysis of this type has limitations. The study was not designed to test for differences between GOLD stages or differences between treatment arms within GOLD stages. The numbers of patients in each stage were different and analyses of treatment subgroups within stages are underpowered. However, the size of the study ensured that the baseline characteristics of patients within each treatment arm was similar in each GOLD stage. TORCH recruited patients with a pre-bronchodilator FEV_1 _of < 60% predicted, but a substantial number of patients fell into GOLD stage II disease, being defined by spirometric severity based on the post-bronchodilator value. It is important to note that all had a clinical diagnosis of COPD and that the mean total SGRQ in stage II patients was 45, indicating that they were a symptomatic group of patients. Finally, we adopted a conservative criterion with respect to study entry based on the former European Respiratory Society reversibility criterion of a change in FEV_1 _of less than 10% predicted. This is likely to have limited our ability to show changes in post-bronchodilator spirometry compared with studies where no reversibility limitation was present [8,14]. However, this is not likely to impact on our data for health status or exacerbations, which are unrelated to reversibility status [12].

## Conclusion

Our data have clinical implications. Although patients in GOLD stage IV have worse outcomes such as health status impairment, higher exacerbation rates and mortality than did those in GOLD stage II, the latter group are not free from these important complications. Secondly, treatment is effective in GOLD stage II as well as in more severe stages of COPD. Finally, the results presented here suggest that patients with COPD may obtain important benefits from SFC combination pharmacotherapy, even at milder stages of disease.

## Competing interests

CRJ has received consulting fees from Altana, AstraZeneca, Boehringer-Ingelheim and GlaxoSmithKline; speaking fees from Altana, AstraZeneca, Boehringer-Ingelheim, GlaxoSmithKline and Novartis; and grant support from GlaxoSmithKline. PWJ has received consulting fees from Almirall, AstraZeneca, GlaxoSmithKline, Novartis, Roche and Spiration; speaking fees from AstraZeneca and GlaxoSmithKline; and grant support from Boehringer-Ingelheim and GlaxoSmithKline. PMAC has received consulting fees from AstraZeneca, GlaxoSmithKline, Nycomed and Pfizer; speaking fees from GlaxoSmithKline and Nycomed; and grant support from Boehringer-Ingelheim and GlaxoSmithKline. BC has received consulting fees from Altana, AstraZeneca, Boehringer-Ingelheim and GlaxoSmithKline; speaking fees from Altana, AstraZeneca, Boehringer-Ingelheim and GlaxoSmithKline; and grant support from Boehringer-Ingelheim and GlaxoSmithKline. JAA is employed by and holds stock in GlaxoSmithKline. GTF has received consulting fees from Boehringer-Ingelheim, GlaxoSmithKline, Novartis and Schering Plough; speaking fees from Boehringer-Ingelheim, GlaxoSmithKline and Pfizer; and grant support from Altana, Boehringer-Ingelheim, Emphasys Medical Inc, Forrest, GlaxoSmithKline, Mannkind Corporation and Novartis. JCY is employed by and holds stock in GlaxoSmithKline. LRW is employed by and holds stock in GlaxoSmithKline. JV has received consulting fees from AstraZeneca, Boehringer-Ingelheim, GlaxoSmithKline, Hoffman-La Roche, Nycomed and Talecris; speaking fees from AstraZeneca, Boehringer-Ingelheim and GlaxoSmithKline; and grant support from GlaxoSmithKline; his wife has been an employee of GlaxoSmithKline and now works for AstraZeneca.

## Authors' contributions

CRJ, PWJ, PMAC, BC, JAA, GTF, JCY and JV contributed to the initiation, design, and conduct of the study, the interpretation of data, and manuscript development; JAA and LRW designed and performed the statistical analyses. All authors have seen and approved the final submitted version of the manuscript.
